# Association of Low Hospital Birth Volume and Adverse Short-Term Outcomes for Neonates Treated with Therapeutic Hypothermia in Rural States

**DOI:** 10.21203/rs.3.rs-5404622/v1

**Published:** 2024-12-18

**Authors:** Alexa Craig, Anya Cutler, Jay Kerecman, Misty Melendi, Leah Marie Seften, Matthew Ryzewski, Allison Zanno, Whittney Barkhuff, Deirdre O’Reilly

**Affiliations:** Barbara Bush Children’s Hospital at Maine Medical Center; MaineHealth Institute for Research; Northern Light Medical Center; Barbara Bush Children’s Hospital at Maine Medical Center; Barbara Bush Children’s Hospital at Maine Medical Center; Elliot Hospital; Barbara Bush Children’s Hospital at Maine Medical Center; Robert Larner, MD College of Medicine, University of Vermont; Robert Larner, MD College of Medicine, University of Vermont

## Abstract

**Objective:**

We hypothesized that outborn neonates from smaller birth volume hospitals would have more frequent adverse short-term outcomes following therapeutic hypothermia (TH).

**Study Design:**

Multicenter retrospective study comparing outcomes for small (<500 births/year), medium (501–1500 births/year), and large (>1500 births/year) hospitals in Northern New England. Multivariable logistic regression assessed the combined outcome of death/severe gray matter injury on MRI, controlling for encephalopathy severity and time to initiation of TH.

**Results:**

531 neonates were included from small (N=120), medium (N=193), and large (N=218) volume hospitals and TH was initiated at a median of 4.5, 4, and 2 hours of life respectively. The odds of the combined outcome were 4.3-fold higher in small versus large birth volume hospitals (95% CI = 1.6, 12.1, p=0.004), but not different in medium birth volume hospitals.

**Conclusion:**

Neonates born in small volume hospitals had significantly higher odds of death or severe gray matter injury following TH.

## Introduction

Timely identification and treatment of neonates who meet criteria for therapeutic hypothermia (TH) poses significant challenges in rural settings, where access to intensive care, neonatal specialists, and diagnostic resources may be limited. The literature is variable regarding whether outborn neonates treated with TH have more frequent adverse outcomes compared to inborn neonates. Assessing this association is challenging due various factors that are linked with being born in a non-tertiary care setting. Outborn neonates are often not resuscitated by clinicians with the same skill set and experience as neonatologists, suggesting that resuscitation practices may play a role. Outborn neonates also must be transferred from the birth hospital to the NICU, which can delay initiation of TH and make early temperature management during transport challenging(1–3). Time to reach target temperature for effective therapy can also vary and occur beyond the six-hour goal(4). Lastly, there may be differences in the overall health and pregnancy care of the mothers delivering in one setting versus the other.

Many studies have commented on the presence or absence of differences in outcomes for neonates born outside a hypothermia center, even if the study was not expressly designed to assess this question. Findings have ranged from studies that report that the majority of deaths occur in outborns(5), to a reanalysis of clinical trial data showing no difference in the benefit of therapeutic hypothermia for those who were inborn versus outborn(6, 7). However, other evidence suggests worse outcomes among outborn encephalopathic neonates with lower rates of seizure free survival(8, 9). Sabsabi et. al concluded that the level of care of the birth hospital in a metropolitan region of Canada significantly impacts outcomes, with neonates born at level one centers experiencing worse outcomes compared to neonates born at level two or three medical centers(10).

Given these conflicting findings, we sought to evaluate our data from four Northern New England tertiary care centers, aiming to reconsider outborn neonates not as a single homogeneous group, but rather as a population with varying levels of risk for adverse outcomes based on the annual birth volume of their hospital of origin. We hypothesized that the risk of short-term adverse outcomes would be higher for outborn neonates transported from small (up to 500 births per year) and medium birth volume hospitals (501–1500 births per year), compared to those born in large volume hospitals (more than 1500 births per year), where TH is performed on-site. The goal of this investigation was to achieve a more nuanced understanding of the risks associated with being born in a rural setting and outcome after TH.

## Methods

### Study Design:

A retrospective cohort study of neonates treated with TH at four tertiary care centers that participate in the Northeast Regional Hypothermia Consortium (see Supplement 1 for details about each institution).

### Primary Outcome:

The primary outcome was in-hospital mortality or severe gray matter injury on brain MRI. Severe gray matter injury was defined by gray matter sub-score ≥9.5 per the Weeke scoring system, found to be 92% sensitive and 95% specific for an abnormal developmental outcome at 2 years defined as death, cerebral palsy (Gross Motor Function Classification System ≥ II), or Bayley Scales of Infant and Toddler Development with scores <85(11). The gray matter injury score was assigned by a team including a neuroradiologist and a pediatric neurologist who reviewed images from all sites.

### Classification of hospitals:

Hospitals were grouped according to annual birth volume. Small hospitals were defined as a birth rate of 1–500 neonates per year, medium hospitals were defined as a birth rate of 501–1500 neonates per year, and large hospitals (tertiary care centers) all had more than 1500 births per year and were also the centers where TH was implemented.

### Environment:

At all four tertiary care centers, TH is employed for neonates who are less than six hours old at the time of initiation, are greater than or equal to 35 weeks gestation, and who have evidence of perinatal asphyxia with moderate or severe encephalopathy categorized by exam during the first six-hours of life. All four institutions maintain the 33–34°C temperature for 72 hours and rewarm over 12 hours with continuous EEG monitoring.

### Study Inclusion/Exclusion Criteria:

**Figure 1:**
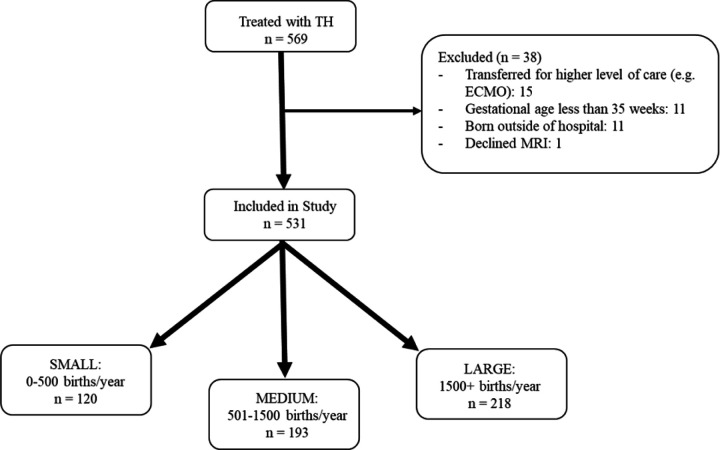
Flow diagram

Neonates were included if they were treated with TH for HIE at any of the four centers. We excluded neonates who were less than 35 weeks’ gestation, completed less than 72 hours of TH, did not undergo brain MRI per family preference, or were transferred to a different institution due to the potential need for extracorporeal membrane oxygenation (ECMO) prior to obtaining a brain MRI. Neonates who were delivered at home or in small birthing centers were also excluded (Figure 1).

### Database:

Data were manually extracted from the electronic medical record by trained research coordinators at each of the tertiary care centers. This study was approved by the institutional review board at each tertiary care center as exempt research.

### Statistical Analysis:

Statistical analysis was conducted using R v.4.2.1. Baseline differences between neonates grouped according to birth volume of hospital in which they were born (small, medium and large) were compared using chi square tests or Fisher’s Exact Tests for categorical variables and t-tests or Kruskal-Wallis rank sum tests for continuous variables. A composite outcome of in-hospital mortality and gray matter injury score ≥9.5 was created to reflect severe outcomes that differ primarily by parental decision making(12, 13). Logistic mixed effects regression analysis was performed to investigate the effect of birth hospital size on the odds of the composite outcome while controlling for possible confounders. Confounders were identified by having a statistical association with both birth volume of the hospital and the composite outcome. Covariates with just an association with the outcome (cutoff of p < 0.2), as well as study site were also included in the full model to improve the precision of estimates. Stepwise selection was performed on the full model using the stepAIC function from the *MASS* package in R to achieve the most parsimonious model. A mediation analysis with 1,000 bootstrapped samples was also performed using the *mediation* package in R to assess the contribution of time to initiation of TH on the difference between outcomes for small versus large birth volume hospitals and medium versus large birth volume hospitals.

## Results

From 2010–2024, a total of 531 neonates were treated with TH across the four participating centers after 38 were excluded (Figure 1). Two hundred and eighteen neonates (41%) were inborn and 313 (59%) neonates were outborn. There were 120 outborn from small birth volume hospitals and 193 from medium birth volume hospitals.

Mothers in small and medium birth volume hospitals were younger and had fewer medical comorbidities (Table 1). Tobacco use was highest for mothers in small birth volume hospitals, and mothers from both small and medium birth volume hospitals had higher rates of marijuana use. There were no significant differences in the rate of Cesarean section or delivery related complications, including placental abruption, cord prolapse, shoulder dystocia, or late fetal heart rate decelerations, however chorioamnionitis and uterine rupture occurred significantly more frequently in the large birth volume hospitals. Neonates in large birth volume hospitals skewed to a younger gestational age (Table 2). The umbilical cord gases demonstrated a greater degree of acidemia for neonates born in medium and large volume hospitals. There were significantly fewer cord gases collected in both small and medium birth volume hospitals. Small birth volume hospitals had the greatest proportions of neonates with mild and severe encephalopathy (Table 2).

The frequency of seizures and severe brain injury on MRI was not statistically significantly different by hospital birth volume (Table 3). Mortality prior to hospital discharge was highest among neonates born at small birth volume hospitals (12%) compared to 3.6% and 6% for medium and high birth volume hospitals, respectively (p=0.008). The composite adverse outcome of in-hospital mortality or severe gray matter injury on MRI occurred for significantly more neonates from small birth volume hospitals (16%) compared to 6% from medium volume and 7% from large volume hospitals (p=0.002). Time to initiation of TH was significantly delayed for both small and medium hospitals, a median of 4.5 and 4 hours, respectively, compared to a median of 2 hours for large volume hospitals (p<0.001).

The full regression model included severity of encephalopathy, time to initiation of TH and the interaction between these two, as well as center where hypothermia was performed, plus additional variables that differed between neonates with and without the primary outcome of death or severe grey matter injury (cesarean section, prolapsed cord, and shoulder dystocia). After stepwise selection, the final model included birth volume and encephalopathy severity. The odds of gray matter injury or death was 4.3 times higher in low birth volume compared to high birth volume hospitals (95% CI = 1.6, 12.1, p=0.004). There was no difference in odds of gray matter injury or death between medium and high birth volume hospitals (p = 0.498). Time to initiation of TH did not mediate the association between small versus large birth volume hospital and the composite outcome (p=0.946).

## Discussion

In this analysis of neonates treated with TH in rural Northern New England, we assessed outcomes for outborns according to the annual birth volume of their hospital of origin. We found that neonates born in small birth volume hospitals compared to large birth volume hospitals have significantly higher odds of death or severe grey matter injury on brain MRI and that the delayed initiation of TH did not contribute to this difference in outcome. Contrary to our hypothesis, the odds of death or severe gray matter injury on brain MRI were not higher for neonates born in medium birth volume hospitals compared to large birth volume hospitals.

Our analysis is the second to consider outborn neonates as subgroups rather than as a homogeneous entity. This type of analysis was first performed by Sabsabi et al who investigated the impact of birthplace (birth in Level I, II or III nursery) on outcome for asphyxiated neonates(10). They found a higher incidence of asphyxia for neonates born in lower care level settings (e.g. level 1 birth centers) compared to Level III centers. In multivariate analysis, birth in a level 1 birth center was associated with a 2-fold higher risk of either death or brain injury. Our results were similar, although the magnitude was higher with 4-fold increased odds of death or brain injury on MRI. One possible reason for the difference in magnitude could be that our population is primarily rural, whereas the Sabsabi study was done in an urban environment(14–16). Another possible reason could relate to the fact that the Sabsabi study was done in a country with nationalized healthcare, a system that has a known association with better outcomes(17).

Small birth volume hospitals are intrinsically different from medium and large birth volume hospitals for several reasons. First, for small birth volume hospitals, the ability to perform an emergency Cesarean section within the standard 30-minute window may not be feasible(18). Small birth volume hospitals are also less likely to have in-house pediatrician coverage to attend deliveries. This may leave the immediate resuscitation of an asphyxiated neonate in the hands of labor and delivery staff members who may have less experience in neonatal resuscitation(19, 20). This is not the case for medium and large birth volume hospitals, which usually have around the clock coverage provided by pediatricians, pediatric hospitalists, or neonatal nurse practitioners who are more experienced in neonatal resuscitation.

In a propensity score-matched analysis of neonates born in the United Kingdom, Shipley et al reported that delivery at hospitals with TH capability was associated with a greater proportion of seizure-free survival (35%), compared to delivery at centers without the ability to provide TH (31% seizure-free survival)(8). Unlike our study, the Shipley et. al. study did not appreciate a significant difference in mortality according to location of birth. This may be attributable to the fact that in the UK, TH can be performed in Level 2 and 3 centers and in this study only 14% of the study population came from Level 1 hospitals, whereas in our study nearly one-quarter of our patients are from Level-1 hospitals. In another large retrospective propensity matched analysis from the Pediatrix group, the authors also did not find a statistically significant higher rate of mortality for outborn neonates(9). However, surviving outborn neonates had significantly higher rates of seizures and gastrostomy tube placement suggesting a difference in morbidity associated with perinatal hypoxia-ischemia. The outcome measures used in these large studies were chosen as available short-term outcomes since longer term outcome measures such as formal neurodevelopmental assessments such as the Bayley Scales of Infant Development (BSID) were not available. Unfortunately, our study also did not have long term data results to analyze neurodevelopmental outcomes after hospital discharge.

Time to initiation of therapeutic hypothermia has been linked to the question of outcome differences for inborn versus outborn neonates. Neonates born outside of a TH center typically have later initiation of TH, which we observed in both small and medium birth volume hospitals in this study. The relationship of earlier time to initiation of TH with improved outcome was first highlighted in preclinical experiments(21–23). In an observational study in humans, Thoresen et. al. demonstrated that surviving neonates with initiation of TH before three hours of life had better motor outcomes at 18–20 months compared to those with initiation after three hours(24). These data, combined with the knowledge that initiation of TH after 6 hours of life had a low probability of benefit(25) resulted in a sense of urgency to initiate TH as quickly as possible.

More recent work has re-assessed this urgency. A retrospective cohort of 91 Canadian born neonates assessed outcome by early (median of 1.4 hours) versus late initiation (median of 4.4 hours) of TH, no differences in the severity of brain injury on MRI or in neurodevelopmental outcomes at 18 months were appreciated(26). A recent secondary analysis of the 500 neonates enrolled in the HEAL Study(27) also did not demonstrate differences in short-term outcomes or 2-year neurodevelopmental outcomes for early (<4 hours) versus late (>4 hours) attainment of the target temperature(28). Time to initiation is confounded by the fact that neonates with severe encephalopathy tend to have TH initiated the soonest, but also have the highest rate of adverse outcome, which was why we chose to use severity of encephalopathy by time to initiation as an interaction term in our regression analysis. Additionally, the timing of the hypoxic ischemic injury onset is often not known with certainty. Our results echo these other clinical studies(26, 28) indicating that time to initiation does not affect outcome.

Our study has several strengths, including a unique consortium, formed by investigators from four NICUs in three of the most rural states in the United States: Maine, New Hampshire and Vermont. This novel consortium has allowed us to collect and analyze data that is not routinely available outside of major academic children’s hospitals. With the combination of four NICU’s, our sample size is large for data sets emerging out of rural areas and may be generalizable to other more rural locations. We used advanced statistical analyses to assess the relationship between outcome and hospital size taking into consideration both severity of encephalopathy and time to initiation of TH, as well as other potential confounders such as site. Additionally, we collected the degree of encephalopathy for all neonates in the study, and our combined outcome captures both mortality and the severe impairment expected in children with high gray matter injury scores on MRI. This combined outcome is key in our opinion as the consequence of either death or severe gray matter injury is most often the result of parental decision making rather than the inability of a neonate to survive(29). Another strength is that we chose to exclude those born in birthing centers and home births which confer a higher risk for adverse outcome(30).

There are several weaknesses in this study, which include the lack of comprehensive data on longer term neurodevelopmental outcomes, and no data on time from birth to target temperature. Additionally, some data on outborn neonates was missing. For example, there were many more missing cord gases for neonates born in both small and medium birth volume hospitals, a challenge reported by others studying TH and outcomes in outborn neonates(3). There is also likely to be some residual confounding from sociodemographic variable differences for deliveries that occur in small and medium birth volume hospitals (e.g. incomplete data on drug exposure, socioeconomic status and prenatal care). This may have resulted in an overestimate of the difference in odds of adverse outcome between small and large birth volume hospitals. Finally, although each institution follows a standardized hypothermia protocol that has strong similarities between institutions, there are likely nuanced differences between institutions that are not captured by the data. We attempted to address this by controlling for site in the stepwise regression model. An additional limitation is the potential for changes in practice across time from 2010–2024. Use of a structured neonatal encephalopathy exam came into more widespread use during the epoch studied and it is very possible that intra-site differences in quality and conclusions of the exam changed during that time, particularly in differentiating mild from moderate encephalopathy.

## Conclusions

This analysis evaluated neonates treated with therapeutic hypothermia from three rural states and separated the neonates that were treated with TH into subgroups based on the birth volume of the hospital of origin. We found that births in small compared to large birth volume hospitals have significantly higher odds of death or severe gray matter injury on MRI. This was not the case for medium volume hospitals, and our results did not show an effect of time to initiate TH on outcome. Further analysis is needed to delineate the obstetric and neonatal resuscitation factors that may be involved in our finding of increased morbidity and mortality associated with lower birth volume hospitals in these three rural states. Potential strategies to support neonatal resuscitation and stabilization may be needed in these small, often critical access rural community hospitals. The use of telehealth modalities may facilitate interactions between expert subspecialists and primary care providers in these efforts to improve outcomes.

## Supplementary Material

Supplement 1

## Figures and Tables

**Table 1:** Characteristics of Birth Parent and Delivery by Hospital Birth Volume

Characteristic	0–500, N = 120^1^	501–1500, N = 193^1^	1501+, N = 218^1^	p-value^2^
**Maternal Age**	29.0 (23.0,32.2)	30.0 (24.0,33.0)	31.0 (26.0,34.0)	**0.016**
**Gestational Diabetes**	11 (9.2%)	20 (10%)	28 (13%)	0.6
**Preeclampsia/Eclampsia**	11 (9.2%)	12 (6.2%)	29 (13%)	**0.053**
**Multiple Gestation**	1 (0.8%)	1 (0.5%)	12 (5.5%)	**0.003**
**Tobacco**	20 (17%)	16 (8.3%)	17 (7.8%)	**0.021**
**Opioids**	11 (9.2%)	10 (5.2%)	16 (7.3%)	0.4
**SSRIs**	9 (7.5%)	26 (13%)	24 (11%)	0.3
**Benzodiazepines**	1 (0.8%)	4 (2.1%)	2 (0.9%)	0.6
**Marijuana**	20 (17%)	27 (14%)	15 (6.8%)	**0.013**
**Maternal fever**	9 (7.5%)	8 (4.1%)	14 (6.4%)	0.4
**GBS positive**	29 (24%)	32 (17%)	56 (26%)	0.083
**Chorioamnionitis**	5 (4.2%)	7 (3.6%)	32 (15%)	**<0.001**
**Prolonged Rupture of Membranes**	18 (15%)	24 (12%)	29 (13%)	0.8
**Late Decelerations**	17 (14%)	33 (17%)	26 (12%)	0.3
**C-Section**	63 (52%)	98 (51%)	120 (55%)	0.7
**Shoulder Dystocia**	11 (9.2%)	19 (9.8%)	27 (12%)	0.6
**Prolapsed Cord**	3 (2.5%)	7 (3.6%)	6 (2.7%)	0.9
**Placental Abruption**	9 (7.5%)	24 (12%)	18 (8.2%)	0.2
**Uterine Rupture**	0 (0%)	4 (2.1%)	10 (4.6%)	**0.03**

1 Median (IQR); n (%)

2 Kruskal-Wallis rank sum test; Pearson’s Chi-squared test; Fisher’s exact test

**Table 2:** Characteristics of Newborns by Hospital Birth Volume

Characteristic	0–500, N = 120^1^	501–1500, N = 193^1^	1501+, N = 218^1^	p-value^2^
**Gestational Age**	39.00 (38.00,40.00)	39.00 (38.00,40.00)	39.00 (37.00,40.00)	**0.002**
**Gestational Age <37wks**	13 (11%)	18 (9.3%)	33 (15%)	0.2
**Birth Weight (kg)**	3.40 (3.00, 3.75)	3.35 (2.96, 3.73)	3.27 (2.89, 3.67)	0.2
**Male Sex**	65 (54%)	109 (56%)	125 (57%)	0.9
**APGAR 1 min**	2 (1, 3)	2 (1, 3)	2 (1, 3)	0.2
Not documented	1	0	0	
**APGAR 5 min**	4. (3, 5)	5 (3, 7)	5 (3, 6)	**<0.001**
Not documented	1	0	0	
**APGAR 10 min**	6 (4, 7)	6 (5, 8)	6 (5, 7)	**0.008**
Not documented	15	22	36	
**Arterial Cord pH**	7.15 (7.02, 7.27)	7.08 (6.96, 7.20)	7.07 (6.94, 7.20)	**0.005**
Not collected	59	58	39	
**Venous Cord pH**	7.23 (7.07, 7.28)	7.19 (7.07, 7.29)	7.22 (7.08, 7.29)	>0.9
Not collected	70	64	36	
**Severity of HIE**				**0.009**
Mild	27 (22%)	34 (18%)	24 (11%)	
Moderate	76(63%)	146 (76%)	169 (78%)	
Severe	17 (14%)	13 (6.7%)	25 (11%)	

1 Median (IQR); n (%)

2 Kruskal-Wallis rank sum test; Pearson’s Chi-squared test; Fisher’s exact test

**Table 3:** Short-term Outcomes of Newborns by Hospital Birth Volume

Characteristic	0–500, N = 120^1^	501–1500, N = 193^1^	1501+, N = 218^1^	p-value^2^
**Death**	15 (12%)	7 (3.6%)	13 (6.0%)	**0.008**
**Severe grey matter injury**	8 (8%)	7 (6%)	8 (7%)	0.7
**Death or severe grey matter injury**	19 (16%)	11 (6%)	15 (7%)	**0.004**
**Seizure**	30 (25%)	53 (27%)	46 (21%)	0.3
**Time to TH Initiation in hours (IQR)**	4.50 (3.00,5.67)	4.00 (3.00,5.00)	2.00 (1.00,3.50)	**<0.001**

1 Median (IQR); n (%)

2 Kruskal-Wallis rank sum test; Pearson’s Chi-squared test; Fisher’s exact test

## Abbreviations

EEG: Electroencephalogram
HIE: Hypoxic Ischemic Encephalopathy
MMC: Maine Medical Center
MRI: Magnetic resonance imaging
NE: Neonatal Encephalopathy
NICU: Neonatal Intensive Care Unit
NLEMMC: Northern Light Eastern Maine Medical Center
TH: Therapeutic hypothermia
UVMMC: University of Vermont Medical Center
