# Supplementary material for: Association of Low Hospital Birth Volume and Adverse Short-Term Outcomes for Neonates Treated with Therapeutic Hypothermia in Rural States
Source: Res Sq. 2024 Dec 18:rs.3.rs-5404622. Preprint. [Version 1] doi: 10.21203/rs.3.rs-5404622/v1 (PMC11702793; doi:10.21203/rs.3.rs-5404622/v1)
Supplement: Supplement 1 [file NIHPPRS5404622v1-supplement-1.pdf]

Supplementary Files

This is a list of supplementary files associated with this preprint. Click to download.

- SupplementalTable111.5.24.docx
